# Rational Development of IT-SOFC Electrodes Based on the Nanofunctionalization of La_0.6_Sr_0.4_Ga_0.3_Fe_0.7_O_3_ with Oxides. Part 2: Anodes by Means of Manganite Oxide

**DOI:** 10.1021/acsaem.2c02592

**Published:** 2022-12-28

**Authors:** Jonathan Cavazzani, Andrea Bedon, Giovanni Carollo, Mathilde Rieu, Jean-Paul Viricelle, Antonella Glisenti

**Affiliations:** † Department of Chemical Sciences, University of Padova, Via F. Marzolo 1, 35131 Padova, Italy; ‡ Mines Saint-Etienne, Univ. Lyon, CNRS, UMR 5307 LGF, Centre SPIN, F − 42023 Saint-Etienne, France; § ICMATE - Department of Chemical Sciences, University of Padova, Via F. Marzolo 1, 35131 Padova, Italy

**Keywords:** manganite oxides, LSGF, perovskite, anode, SOFC, propane, hydrogen

## Abstract

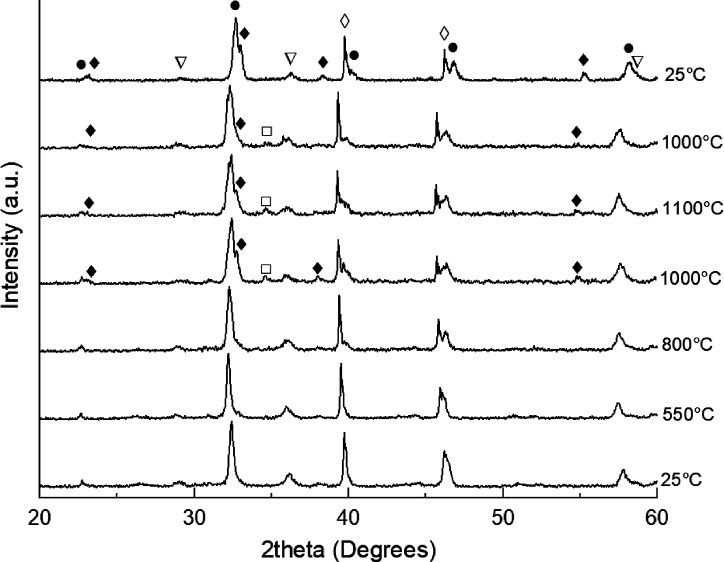

To promote the diffusion on the market of solid oxide fuel cell (SOFC) devices, the use of fuels other than the most appealing hydrogen and also decreasing the working temperature could show the way forward. In the first part, we concentrated our efforts on cathodes; hereby, we focused on anodes and concentrated our efforts to develop a sustainable multifuel anode. We decided to develop LSGF (La_0.6_Sr_0.4_Ga_0.3_Fe_0.7_O_3_)-based nanocomposites by depositing manganite oxide to enhance the performance toward propane. MnO_x_ has been deposited by a wet impregnation method, and the powders have been largely characterized by X-ray diffraction, scanning electron microscopy, energy-dispersive X-ray analysis, X-ray photoelectron spectroscopy, hydrogen temperature-programmed reduction, oxygen temperature-programmed desorption, and N_2_ adsorption. Cell performances were first collected in hydrogen as a function of both the temperature and hydrogen content. EIS measurements were studied using Nyquist and Bode plots, and they show two processes at high frequency, assigned to charge transfer at the electrode/electrolyte interface, and at low frequency due to the dissociative adsorption of hydrogen. The Arrhenius plot of area specific resistance suggests two different trends, and the activation energy decreases from 117 kJ/mol at 750 °C to 46 kJ/mol above that temperature. This behavior is often connected to chemical modification of the catalyst or changes in the limiting step processes. Power densities in hydrogen and propane were determined at 744 °C after 1 h of operation, achieving 70 mW/cm^2^ in H_2_ and 67 mW/cm^2^ in C_3_H_8_. The open-circuit voltage increases from 1.10 V in hydrogen to 1.13 V in propane.

## Introduction

The energy transition and the tendency to reduce the environmental footprint from energy production are pushing toward scenarios with great opportunities for solid oxide cells (SOCs) that must be exploited.^1,2^ Solid oxide fuel cells (SOFCs) allow high efficiency for the electricity production from fuels (up to 90%),^3,4^ so they can be fundamental during the transition to renewable sources as they can produce the same amounts of energy as conventional generators with half consumption of fuel. Hydrogen represents the most suitable and appealing fuel for feeding this technology since only water is produced as waste.^5,6^ However, the problems raised by hydrogen supply, storage, and transportation break the commercial development of SOFCs.^7,8^ To accelerate the commercial development of SOFCs, intermediate steps are required. One of them may be the development of SOFCs that can operate directly with hydrocarbons, which possess an already well-developed supply service. Traditional Ni–YSZ cermets are recognized as the state-of-the-art material when operated on hydrogen^9−13^ owing to the unbeatable trade-off between the low cost of the materials and their great performances. However, the activity of Ni toward the formation of carbon leads to the poisoning of the anode by coking, affecting long-term operation.^14−16^

To remediate these significant breaks, the scientific community is pushing efforts in the development of new anodic materials that could allow a commercial rise of the SOFC technology operating on hydrocarbons.^17−22^ A class of material consists in mixed ionic electronic conductive materials that fulfill the main requirement of anodic materials attracts attention. To enhance the catalytic activity of these ceramic materials, nano-structuration processes are employed to increase the electrocatalytic activity while using a minimal quantity of metals. Traditional nano-structuration processes include impregnation and infiltration, which consist in the deposition of the nanocatalysts via a wet-chemistry procedure.^23−25^

In the first part of this work,^26^ we studied modified LSGF (La_0.6_Sr_0.4_Ga_0.3_Fe_0.7_O_3_) impregnated by FeO_x_ to promote oxygen reduction at the cathode in SOFC technology. Hereby, we maintained LSGF perovskite but also provided it with functionalities for working as an anode using hydrogen and propane.^27−30^

It is well known that LSGF-based materials exhibit good stability in both environments and good electrical and ionic conductivity due to iron oxidation states promoted by the doping of strontium and lanthanum in the A-site. Meanwhile, Ga in the B-site leads to excellent stability in a reducing atmosphere.^31,32^

Tailored activity toward hydrocarbons has been provided by MnO_x_ impregnated on LSGF. As a starting point, LSGF is known to show activity toward methane oxidation,^33^ a characteristic shared by ferrite-based materials.^34,35^ Mn-based oxides have been extensively studied for the oxidation of hydrocarbons.^36−40^ Baldi *et al.*(41) studied the activity of α-Mn_2_O_3_ and Mn_3_O_4_ for propane oxidation and spinel-type phase Mn_3_O_4_ exhibits a slightly higher activity than α-Mn_2_O_3_. In addition, Chen *et al*.^42^ studied the behaviors of MnO_2_ and doped it by Ni for propane conversion. Their data exhibits that the C_3_H_8_ oxidation reaches 90% at 245 °C for undoped MnO_2_. These works suggest that many manganese oxides can contribute to propane oxidation during fuel cell operation.

The impregnated LSGF + MnO_x_ powders have been characterized through X-ray diffraction (XRD), hydrogen temperature-programmed reduction (H_2_-TPR), X-ray photoelectron spectroscopy (XPS), and energy-dispersive X-ray analysis (EDX). Surface modification subsequent to oxide deposition has also been studied by N_2_ adsorption isotherms, oxygen temperature-programmed desorption (O_2_-TPD), and scanning electron microscopy (SEM) images. The electrochemical performances were evaluated by means of electrochemical impedance spectroscopy (EIS). Because the deposition of electrodes involves treatment at high temperatures that are potentially able to modify the LSGF composite, the powder has been treated under the same conditions and thoroughly examined to monitor closely any modification of the material consequent on each preparation step. The behavior of MnO_x_/LSGM was first evaluated in hydrogen, performing electrochemical measurements using a complete cell. A commercial cathode was used to make its contribution negligible. Then, we demonstrate that La, Sr, and Ga-based perovskite can be used in all components of SOCs because of its stability under both working conditions.^43^ Indeed, button cells were prepared by depositing the above nanocomposites (electrodes) on the LSGM (La_0.9_Sr_0.1_Ga_0.8_Mg_0.2_O_3_) electrolyte. LSGM being a perovskite with a composition very similar to LSGF, we expect a very good ionic conductivity while keeping electronic and hole conduction negligible.^44−46^ The extreme similarity between the structure and composition of an LSGF electrode and an LSGM electrolyte should avoid major solid-state reactions leading to the degradation of the device because of the formation of secondary insulating phases. The final design of the complete cell was chosen as follows: MnO_x_ + LSGF|LSGM|LSGF + FeO_x_, and it reaches power densities of 70 mW/cm^2^ in hydrogen and 67 mW/cm^2^ after 1 h of operation at 744 °C. We demonstrated with this work and with the one focused on cathodes that an SOFC in which electrodes and electrolytes are very similar can be successfully demonstrated and LSGF can be a good support for both the anode and cathode. However, other efforts and improvements, such as thinner electrolytes or metal infiltration to form a homogeneous electron pathway,^47^ would bring important benefits to the anode for this application.

## Experimental Section

### Synthesis: Nanocomposite Powder Synthesis and Treatment

The supporting LSGF perovskite was prepared via solid combustion synthesis.^48^ LSGF was then added to a solution of manganese(II) acetate, and the mixture was kept under stirring for 24 h. The amount of Mn(II) vs supporting perovskite was selected to obtain 10 and 30% mol deposition. The suspension was then heated to eliminate the solvent by evaporation. The resulting powder was treated at 550 °C to remove the organic fraction. The impregnated powders were than calcined at 1000 and 1100 °C, the same temperatures for preparing the solid oxide cells.

### SOFC Preparation: Button Cell Manufacturing

Cells for EIS measurements were electrolyte-supported and prepared by screen printing the LSGF (La_0.9_Sr_0.1_Ga_0.8_Mg_0.2_O_3_)-based material to be tested on both sides of an LSGM pellet (diameter: 20 mm, thickness: 1.2 mm). The pellet was prepared by pressing the LSGM powder (commercial LSGM8282, Treibacher) and treating the so-obtained pellet at 1500 °C for 10 h. Ink preparation involved mixing LSGF impregnated 10% mol MnO_x_ powder and adding the binder V400 (ESL, commercial) and solvent T404 (ESL, commercial) to adjust viscosity. The ink deposition was carried out by means of a homemade screen-printing machine. The layer was then dried in an oven at 100 °C for 15 min. Two round layers with area on each side of the LSGM pellets were printed in this way. In 10% mol MnO_x_/LSGF|LSGM|LSCF full cells (commercial LSCF, lanthanum strontium cobalt ferrite powder, La_0.60_Sr_0.40_Co_0.20_Fe_0.80_O_3_, Sigma-Aldrich), electrodes have areas about 1.26 cm^2^. The MnO_x_/LSGF electrode was treated at 1100 °C to ensure a good electrical contact between the electrolyte and the electrodes. Treatments at temperatures lower than 1000 °C have been found not to be sufficient to ensure enough mechanical stability to the electrodic layer. LSCF electrodes were treated at 1000 °C for 2 h. In 10% mol MnO_x_/LSGF|LSGM|10% mol FeO_x_/LSGF cells, the anode was prepared as barely above while FeO_x_/LSGF was treated at 1000 °C for 6 h as suggested by the first part of this work.^26^ In this case, electrodes have areas about 1.07 cm^2^. Finally, a gold grid was printed from a gold ink (8880-H, ESL, commercial) on both the electrodes, and the cell was treated for the last time at 800 °C for 2 h.

### Characterization

#### XRD

The *in situ* XRD analyses at high temperatures were carried out with a Bruker D8 Advance diffractometer with Bragg–Brentano geometry using Cu Kα radiation (40 kV, 40 mA, λ = 0.154 nm) equipped by a high temperature chamber HTK16 (Anton Paar) and a scintillation detector preceded by a graphite monochromator. The *ex situ* XRD analyses were performed with a Siemens D5000 diffractometer equipped with a rotating platinum sample holder. The data were collected at 0.03° in the (2θ) range from 20 to 60°. The crystalline phases were identified by the search-match method using the JCPDS database.

#### SEM

Field emission-scanning electron microscopy and EDX measurements were carried out using a Zeiss SUPRA 40VP. EDX analyses were set with acceleration voltages at 20 kV (Supporting Information, Figure SI.3).

#### H_2_-TPR and O_2_-TPD

Temperature-programmed reduction (TPR) and oxygen temperature-programmed desorption (O_2_-TPD) were carried out with an Autochem II 2920 Micromeritics, equipped with a thermal conductivity detector (TCD). The measurements were led in a quartz reactor by using 50 mg of sample and heating from RT to 900 °C at 10 °C min^–1^ under a 50 mL min^–1^ constant gas flow. The gas mixture was 5% H_2_ in Ar for H_2_-TPR and Ar for O_2_-TPD. TPR samples were previously outgassed with He (50 mL min^–1^) at room temperature. O_2_-TPD samples were previously fully oxidized at 900 °C under pure O_2_ for 2 h. Measured gas volumes were determined by calibrating the TCD with proper standards and then converting to the molar amount reported in Tables 2 and 3. An ESS Evolution mass quadrupole was used to check that only oxygen was released during O_2_-TPD measurements.

#### N_2_ Adsorption Isotherms

Isotherms were measured at 77 K with a Micromeritics ASAP 2020 plus instrument. The superficial area by the BET model and porosity by DFT calculations were calculated from adsorption curves (Supporting Information, Figure SI.4).

#### XPS

The XPS measurements were carried out with a Perkin Elmer 5600ci Multi Technique System. The spectrometer was calibrated by assuming the binding energy (BE) of the Au 4f7/2 line to be 84.0 eV with respect to the Fermi level. Both extended spectra (survey—187.85 eV pass energy, 0.5 eV step^–1^, 0.05 s step^–1^) and detailed spectra (for La 3d, Fe 2p, Ga 2p, Sr 3d, Mn 2p, O 1s, and C 1s—23.50 eV pass energy, 0.1 eV step^–1^, 0.1 s step^–1^) were collected with a standard Al Kα source working at 250 W. The standard deviation in the BE values of the XPS line is 0.10 eV. The atomic percentage, after a Shirley-type background subtraction,^49^ was evaluated by using PHI sensitivity factors.^50^ Repeated measurements have revealed that the error can be estimated in the first decimal place (and thus no decimals have been reported in the atomic composition). The peak positions were corrected for the charging effects by considering the C 1s peak at 285.0 eV and evaluating the BE differences.^51^ The fittings have been carried out by means of XPSPEAK software, considering a Shirley background and Voight functions for the components. The same full width at half maximum and % of Lorenzian have been used for all the components in a signal.

#### EIS

Electrochemical impedance spectroscopy (EIS) tests were performed on the following complete cells: 10% mol MnO_x_ + LSGF|LSGM|LSCF and 10% mol MnO_x_ + LSGF|LSGM| LSGF + 10% mol FeO_x_. Cell tests were carried out in hydrogen and in propane. While the cathode was fed by air (normal operating conditions for a double cell SOFC cathode). Data were collected with Solartron 1286 Electrochemical Interface and the Solartron 1255 Frequency Response Analyzer devices. The applied excitation voltage is 20 mV.

## Results and Discussion

### *Ex Situ* vs *In Situ* XRD

The results of the analyses on LSGF have been already described in the first part of this work,^26^ and it will not be repeated here. In most cases, data about pure LSGF has been maintained to evidence the modifications brought by the deposition of the manganese oxides. To monitor the evolution of manganese phases during thermal treatments, a series of XRD measurements have been performed *in situ* during heating from 25 to 1100 °C. The aim is to study the behavior and to observe any modifications undergone by the electrode powders while preparing the SOFC as the chosen thermal treatment is the same as cell firing (1100 °C). Using 10% mol of MnO_x_, no further phases beyond LSGF perovskite was observed (Figure SI.1); in any case, these measurements remark the stability of the LSGF phase: no variation in its signals was detected during the whole process. To overcome the poor detectability of Mn oxide phases and to enable a more accurate study, the same test has been repeated also on a 30% mol Mn composite (prepared only for this experiment) to foresee and go into depth of the interaction between LSGF and manganese oxide. All the other tests described in this paper have been, however, performed using 10% mol MnO_x_ on LSGF.

**Figure 1:**
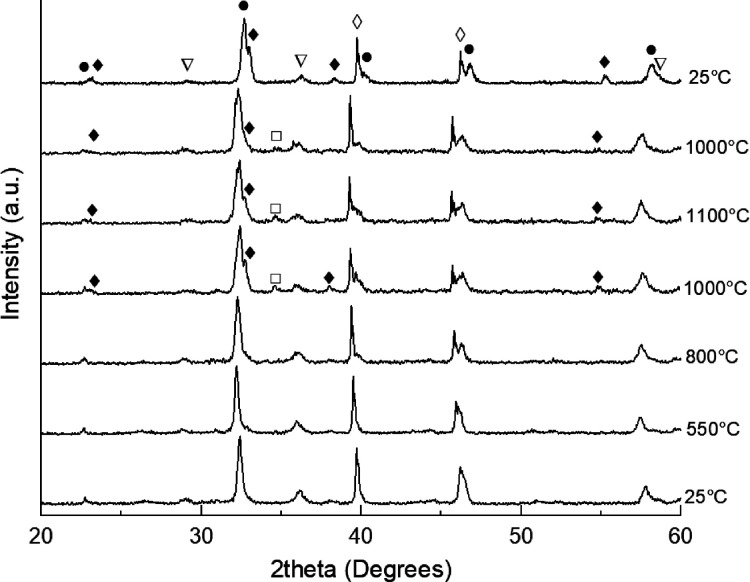
20–60° (2theta) XRD patterns of 30% mol MnO_x_ on LSGF perovskite, treated from 25 °C (lowest pattern) to 1100 °C and then back to 25 °C (highest pattern). solid circles: LSGF, rhombohedral, JPCDS 04-016-7460; open squares: Mn_3_O_4_, cubic, JPCDS 04-002-5633; open inverted triangles: Mn_3_O_4_, hausmannite, tetragonal, JCPDS 04-007-1841; solid diamonds: Mn_2_O_3_, bixbyte, cubic, JPCDS 00-041-1442; open diamonds: Pt (substrate), cubic, JPCDS 00-004-0802.

*In situ* XRD measurements on 30% MnO_x_/LSGF are reported in Figure 1, left. The 25 °C pattern before temperature treatment noteworthily shows the presence of a Mn_3_O_4_ hausmannite phase, which normally forms above 1000 °C^52^ (but the sample has been treated only at 550 °C in this case); the presence of the perovskitic phase plays probably a role in stabilizing phases that would form at higher temperatures. This could be of some interest because Mn_3_O_4_ itself is studied for its catalytic activity.^36,53^ Manganese oxide reflections are also characterized by some broadness, an indication of the small size of the crystallites; the high dispersion could be a reason for the absence of MnO_x_ signals in the 10% composite pattern (Figure SI.1).

Above 1000 °C, during heating, the signals of the pristine hausmannite phase Mn_3_O_4_ decrease in intensity. At the same temperature, two new manganese phases appear: Mn_2_O_3_, normally forming below 1000 °C, and a cubic Mn_3_O_4_. Also, in this case, the contribution of LSGF is clearly determined for stabilizing the manganese oxides. At 1100 °C and also 1000 °C during cooling, the intensity of the Mn_2_O_3_ reflections decreases; this behavior may be assigned to a diminishing of the Mn_2_O_3_ phase because a part of Mn atoms has been incorporated in the perovskite structure. Therefore, only the most intense reflection (at 32.8°) is visible for Mn_2_O_3_ in Figure 1. During the cooling phase, no further modification of the reflections of the hausmannite is observed, while after the complete cooling down, the Mn_3_O_4_ phase totally disappeared. During the thermal treatment, a shift toward lower angles occurs, although no other manganese phase is observed to clearly decrease or disappear, except for Mn_2_O_3_ at the highest temperature. This behavior suggests that a small part of manganese atoms has been embedded inside the perovskite lattice since manganite and iron have similar sizes (respectively, 0.58 and 0.55 Å with VI coordination and 3+ oxidation state) with Mn^3+^ a bit larger than Fe^3+^. The incorporation of Mn^3+^ atoms causes an expansion of the lattice, and it justifies the shift at lower angles of the patterns above room temperature. However, during the cooling down, the manganite atoms have been expelled from the lattice because the final position of the reflection in the LSGF pattern corresponds to the initial ones. In addition, from 1000 °C, the main reflection at 32–33° (2theta) shows a shoulder at lower angles: it can be assigned to a phase transition from rhombohedral perovskite to Brownmillerite, featured by a doublet in the most intense reflection of the crystal structure, due to the further embedded oxygen in the structure during the thermal treatment. The Brownmillerite structure is preserved after the cooling down at room temperature. To support these considerations, H_2_-TPR measurements, described below, exhibit that in reducing conditions (5% H_2_/Ar), there is not any shoulder in the main reflection because no further oxygen can be incorporated in the lattice.

Finally, some data from H_2_-TPR seem to suggest that manganese can also segregate at the grain boundaries. In any case, XPS quantitative analyses (Table 1) indicate that there is some interaction between LSGF and overlaying manganese oxide, as described in the XPS and EDX Characterizations Section. XRD data prove that this interaction, which comprises exchange of atoms and variations of the manganese oxides phase, is continuous; the LSGF and MnO_x_ phases respond jointly at each temperature and show a complex behavior. It is also worth noting that the system LSGF + MnO_x_ evolves depending on temperature, and cooling does not simply “freezes” the crystalline phases.

**Table 1:** XPS and EDX Abundances^a^

sample		La	Sr	Fe	Ga	O	Mn	La/Sr	Fe + Ga/La + Sr	Ga/Fe	Mn/Ga + Fe	Fe + Ga/La
LSGF	Nom	12	8	14	6	60		1.5	1.0	0.4		1.7
		30	20	35	15							
	XPS	4	13	9	3	71		0.3	0.6	0.4		2.5
		15	46	28	11							
	EDX	13	8	15	6	58		1.7	1.0	0.4		1.6
		31	18	36	15							
LSFG 1000 °C	XPS	4	11	6	3	76		0.4	0.5	0.5		2.0
		18	47	23	12							
LSFG 1100 °C	XPS	6	9	7	3	75		0.7	0.7	0.4		1.6
		24	36	28	12							
LSGF + MnOx	Nom	12	8	13	6	60	2	1.5	1.0	0.4	0.1	1.6
		29	20	32	15		5					
	XPS	3	5	6	4	72	11	0.7	1.2	0.7	1.2	2.8
		12	16	20	13		39					
	EDX	12	7	14	5	60	2	1.6	1.0	0.4	0.1	1.6
		29	18	34	14		6					
LSGF + MnOx 1000 °C	XPS	6	7	8	7	72	1	1.0	1.1	0.9	0.1	2.3
		22	23	27	25		4					
LSGF + MnOx 1100 °C	XPS	5	10	5	5	74	1	0.5	0.7	0.9	0.1	2.1
		18	40	20	19		3					

fitting results							
	O 1s				Mn 2p_3/2_		
	O1	O2	O3	O4	Mn1	Mn2	>Mn3
LSGF + MnOx	527.6	529.5	531.0	532.4	640.3	641.8	643.4
	11	39	26	24	25	46	29
LSGF + MnOx 1100 °C	527.8	529.2	530.7	532.0	640.9	641.9	643.2
	8	33	42	16	25	41	34

a Composition (at %) of LSGF and LSGF + MnO_x_ before and after 1000 °C/1100 °C treatments as measured by means of XPS and EDX. The nominal (Nom) compositions are reported for comparison. The composition (at %) considering only cations is in the second row. The peak position and relative amount obtained by the fitting procedure applied to O 1s and Mn 2p_3/2_ signals are also reported.

### XPS and EDX Characterizations

The EDX compositions agree with the ones expected from the weighted amounts (nominal compositions), but significant differences are observed in the outer layer composition (XPS) as a consequence of the deposition and heating treatment. In MnO_x_/LSGF, the oxide phase is deposited on the surface of the perovskite and no significant diffusion inside the perovskite is observed in the as-prepared composite; this is proved by the Mn/(Fe + Ga) atomic ratio higher than the nominal one on the surface (XPS results). Composites show a more complex behavior when heated at 1000 °C or more: deposited cations diffuse into the perovskite, and their XPS concentration decreases (the Mn/(Fe + Ga) atomic ratio reaches the expected value). This trend was previously suggested by XRD data. In perovskites, frequently, A-site cations surface segregate; when both La and Sr are present in the oxide, the surface segregating cation can change depending on composition: in LSGF, Sr surface segregates; the thermal treatment promotes the La migration toward the surface. After deposition of manganese oxide, the surface segregation is modified and the La amount increases; the thermal treatments induce again the Sr surface segregation. It is interesting that manganese seems to expel gallium from the inner to the surface: the atomic percentage (at %) of Ga increases after the treatment at 1000 °C as well as the Ga/Fe atomic ratio. In the LSGF + MnO_x_ composite, the Mn 2p peaks’ position and shape (see the Supporting Information, Figure SI.5) are consistent with the presence of the following components: Mn(II) (640.3–640.9 eV), Mn(III) (641.8–641.9 eV), and Mn(IV) (643.2–643.4 eV)^50,54^ (Table 1). The thermal treatments cause the increment of the relative amount of Mn(IV). Interesting differences are also observed in the O 1s signals. Also, in this case, the peak shape is consistent with different species: perovskite lattice oxygen (527.6–527.8 eV), oxygen bound to transition metals (529.2–529.5 eV), oxygen bound to non-transition metals (530–530.5 eV), and oxygen of hydroxides.^54,55^ After the thermal treatments, the component at around 529 eV decreases, whereas the one at about 530 eV increases: this is consistent with the decrease in the amount of Mn due to the diffusion inside the bulk and the increment of Sr.

### H_2_-TPR

**Figure 2:**
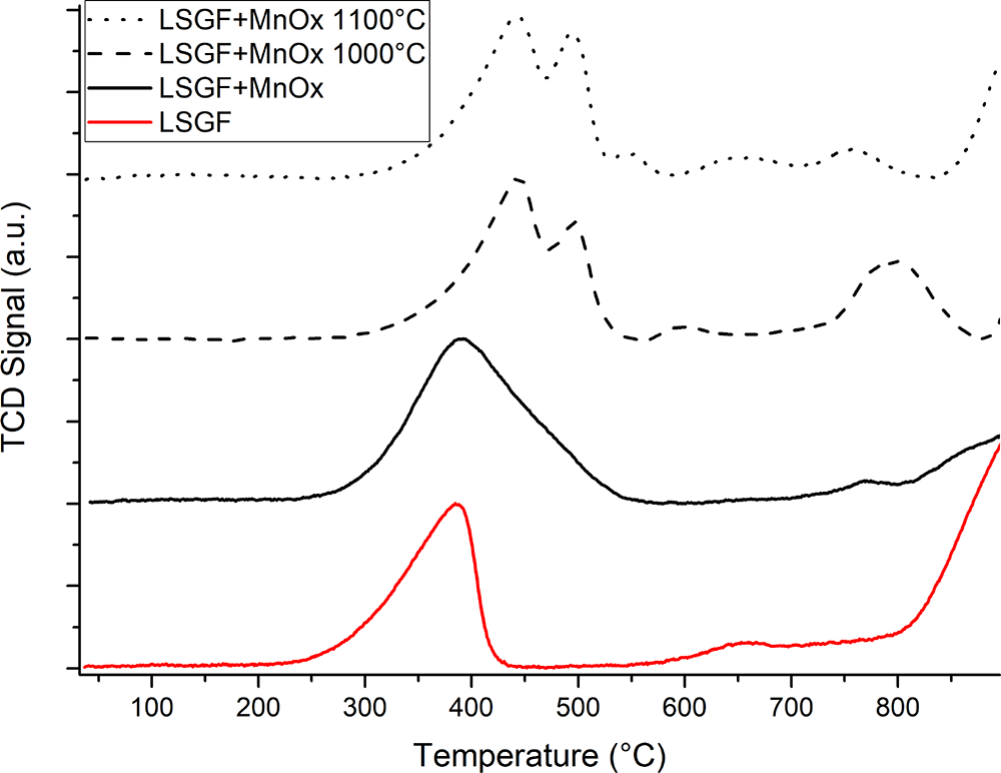
H_2_-TPR profiles of LSGF + MnO_x_ treated at different temperatures. In red, the profile of LSGF before the deposition is shown for comparison.

Figure 2 shows the TPR profile of the LSGF and LSGF + MnO_x_ powders. Compared to pure LSGF, samples with manganese oxides show several signals superimposed to the LSGF ones. Stobbe *et al*.^56^ report that the reducibility of manganese oxides has been found to significantly depend on the specific surface area of the particles. In their work, MnO_2_ showed almost a one-step reduction to Mn(II) around 500 °C, whereas Mn_2_O_3_ showed a clear two-step reduction (around 300 and 400 °C): from Mn_2_O_3_ to Mn_3_O_4_ and then to MnO. Kapteijn *et al*.,^57^ in contrast, observed a two-step reduction for MnO_2_ (the reduction temperature of Mn^4+^, 331–351 °C, being lower than that of Mn^3+^, 443–526 °C).^59^ A literature data comparison suggests a deep influence of the preparation procedure, sample history, and crystalline degree on the TPR behavior.

The as-prepared LSGF + MnO_x_ sample (Figure 2 and Table 2) shows a broad band between 300 and 500 °C. The contribution around 350–400 °C is due to the reduction Fe^4+^ → Fe^3+^ of the perovskitic iron in the LSGF lattice, the one around 450 °C to manganese. The hydrogen consumption of this last peak is estimated (by subtracting the expected 115 mmol/mol consumption for the perovskite contribution determined in the unsupported LSGF) to be around 35 mmol/mol. According to nanocomposite structural data interpretation (XRD, Figure 1), the manganese oxide is likely to be deposited as Mn_3_O_4_: the Mn^3+^ → Mn^2+^ reduction of this phase to MnO is compatible with the calculated area, so confirming the validity of the hypothesis. The temperature also supports this hypothesis being in the range already mentioned for Mn^3+^ reduction. No signal indicating further reduction to Mn^0^ is observed in the examined temperature interval.

**Table 2:** Hydrogen Consumption (mmol/mol) from H2-TPR Peaks^a^

sample	400 °C	500 °C	800 °C
LSGF	115	0	0
theoretical LSGF + 10% Mn_3_O_4_	145		0
LSGF + MnOx	150 (sum of two peaks)		traces
LSGF + MnOx 1000 °C	100	35	40
LSGF + MnOx 1100 °C	55	35	5

a Hydrogen consumption for main peaks in Figure 2 (mmol/mol of perovskite). Brackets indicate deconvolution of groups of peaks.

After the treatment at 1000 °C, the area of the Fe^4+^ → Fe^3+^ reduction around 400 °C of the perovskite slightly decreases (Fe^4+^ fractions are 29% after the deposition and 33% before), while the intensity of the peak around 450–500 °C assigned to manganese Mn^3+^ → Mn^2+^ does not seem to be modified, although it is less broad. It is relevant to observe that this Mn^3+^ → Mn^2+^ peak can include, other than the Mn_3_O_4_ contribution, a Mn_2_O_3_ fraction that was indicated to form by XRD measurements and the eventual fraction of Mn^3+^ that could have entered inside the perovskite lattice: no significant differences among reduction temperature are, in fact, expected. A new peak at 800 °C appears, suggesting the further reduction of manganese: Mn^2+^ → Mn^0^. Literature data indicate that, at this temperature, the reduction of Mn(II) cations can be observed in the perovskite lattice.^58^ The area of this peak is slightly less than half the one expected for the complete reduction of all deposited manganese and this indicates that only a portion of Mn(II) is reduced in this process. Probably, after the thermal treatment at 1000 °C, half of the total deposited manganese is inside the perovskite lattice. Inside the perovskite, Mn would have an oxidation state +3, and the area of the 500 °C peak is consistent with the 800 °C peak for the reduction of the same amount of perovskitic manganese (half of the total Mn amount). The absence of any peak before 350 °C excludes the presence of Mn^4+^.

TPR data, as a whole, confirm that part of the manganese penetrates inside the perovskite cell but do not account for the entire amount of the deposited manganese, which probably have adopted another form. After 1100 °C treatment, the perovskitic Fe^4+^ → Fe^3+^ 400 °C peak further decreases (Fe^4+^ fraction, calculated from peak area, is 16%), and the area of the manganese peak at 450 °C is maintained, whereas that of the peak at 800 °C strongly decreases. This suggests that manganese atoms, which had previously entered into the perovskitic structure, could have been expelled from it. Nonetheless, the Fe^4+^ → Fe^3+^ variation implies that the joint effect of temperature and manganese presence has modified the bulk of LSGF. The ability of Mn to segregate as a defect in Sr vacancies and at grain boundaries with oxidation state 2+ was observed in other perovskites.^59^ This behavior is also consistent with the relevant strontium surface segregation and the decrease in Sr with increasing temperature, agreeing with the observation that only a fraction of deposited manganese is actually observed in the TPR curve (in the +2 state, it would overlap with Fe^3+^ reduction at 800 °C). Furthermore, this accommodation for manganese would not impact on XRD reflections, and in fact, no modification of the position of LSGF is observed in all LSGF + MnO_x_ samples. To reveal the stability of the materials in a reducing atmosphere, we performed XRD measurements after the TPR analysis, reported in the Supporting Information, Figure SI.2. Patterns demonstrate that LSGF exhibits a good stability in a reducing environment because the perovskite structure is preserved. However, a low intensity reflection belonging to iron appears after the TPR treatment with the consequent formation of La_2_O_3_. The formation of lanthanum oxide will probably cause a worsening in the electrochemical performances.

### O_2_-TPD

**Figure 3:**
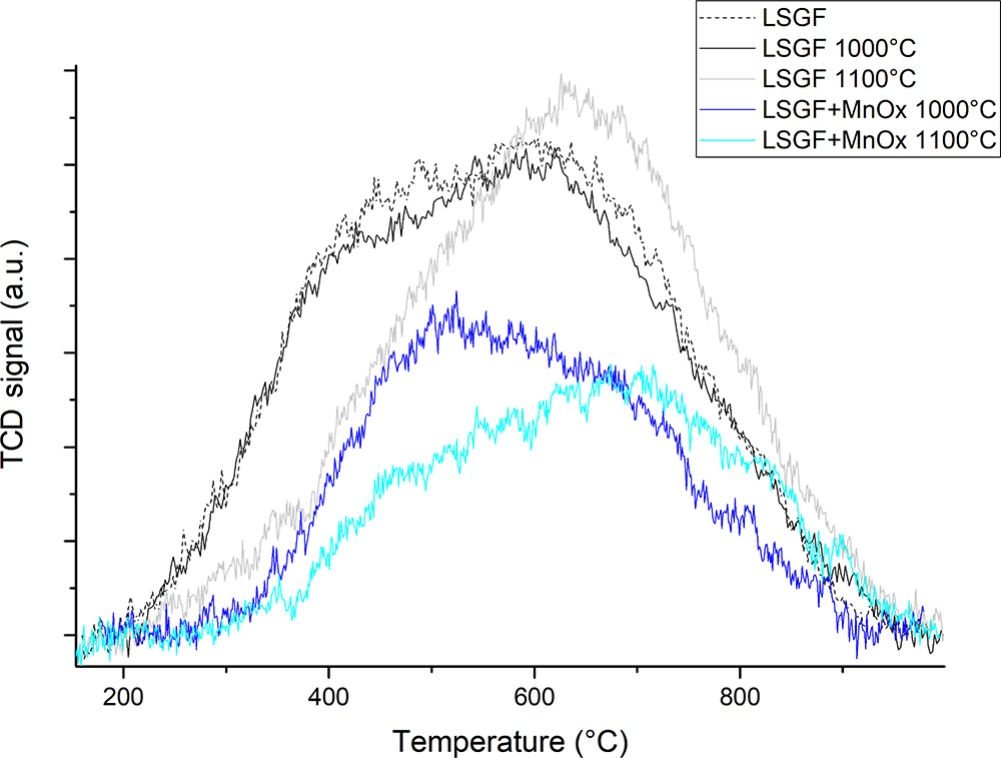
O_2_ desorption of the tested samples. O_2_-TPD profiles of LSGF and LSGF + MnO_x_ treated at different temperatures. The profile of LSGF treated at 900 °C for the calcination is represented by the dashed line.

The results of O_2_-TPD measurements obtained after the thermal treatment at 1000 and 1100 °C for LSGF + MnO_x_, compared with pure LSGF, are shown in Figure 3. The amount of oxygen released is summarized in Table 3. The mass quadrupole indicated that oxygen was the only species in the carrier stream at the instrument outlet. All the curves show an oxygen release that begins at 200–250 °C and does not end until 1000 °C. Conventional separated α and β oxygen peaks (respectively, surface and lattice oxygen) cannot be discriminated; instead, the two types of oxygens produce two broad, not completely resolved, signals.

**Table 3:** Amount per Mole of Desorbed Oxygen during O2-TPD Measurement (mmol/mol of Perovskite)

sample	900 °C	1000 °C	1100 °C
LSGF^a^	52	48	46
LSGF + MnO_x_		26	19

a 900 °C is the calcination temperature of LSGF during its synthesis.

In LSGF + MnO_x_, the decrease in oxygen desorption is more severe, particularly when considering the lattice oxygen. The increasing temperature induces the decrease in the signal around 450–500 °C, suggesting that a lower amount of oxygen active species desorbs from the surface. The reduction of 1/2 of the α contribution can be a rough indication that at least half of the total LSGF surface is covered by other phases. In LSGF + MnO_x_, the amounts of oxygen desorbed are lower; in addition, increasingly less intense TPR signals are observed after the thermal treatments at 1000 and 1100 °C. Fe^4+^ and oxygen vacancies are generated to balance charge neutrality when Sr, limited to oxidation state +2, is inside the perovskitic structure. Therefore, a decrease in both the concentrations of Fe^4+^ and oxygen vacancies can be consistent with the XPS results about strontium. This element segregates on the surface, as it happened with FeO_x_;^26^ in the case of FeO_x_/LSGF, the relevant amount of iron allows the formation of a stable SrFeO_3_ phase. In the MnO_x_/LSGF case, in contrast, the amount of iron is lower and there is a consistent amount of gallium, so the formation of SrFeO_3_ is possible. The progressive depletion of strontium, triggered in some way by manganese, explain the H_2_-TPR and O_2_-TPD results.

### Electrochemical Measurements

#### MnO_x_/LSGF|LSGM|LSCF Full Cell

**Figure 4:**
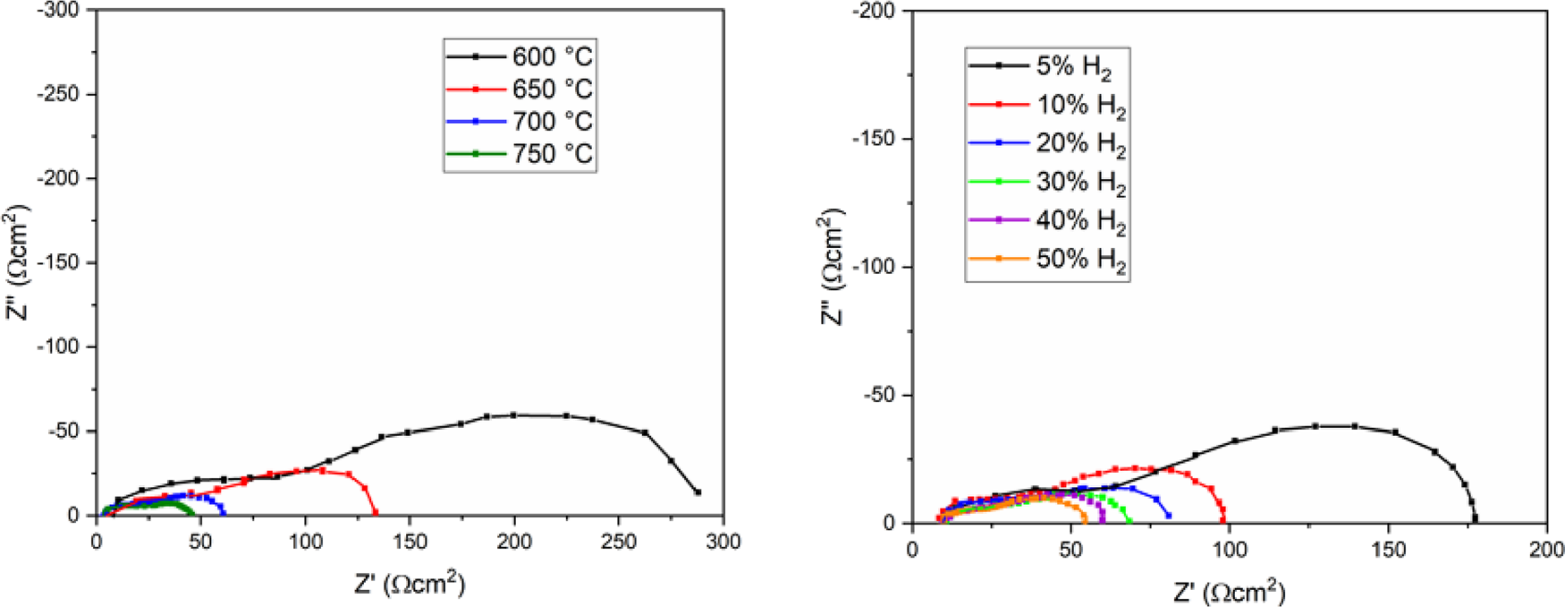
Nyquist plots of the full cell MnO_x_/LSGF|LSGM|LSCF are reported as a function of temperature using 30% of hydrogen (left) and as a function of hydrogen content at 700 °C (right).

The EIS responses for the complete cell, where the anode was made by MnO_x_ impregnated on LSGF perovskite and the cathode by commercial LSCF, are shown in Figure 4. Variations depending on temperature are reported in Figure 4 left while depending on hydrogen concentration in Figure 4 right. Two processes are well distinguishable, namely, high frequency (HF) and low frequency (LF), depending on their characteristic frequencies. Bode plots are reported in the Supporting Information (Figure SI.6). Both processes are well fitted by a resistance in parallel with a CPE. More information about data fitting are reported in the Section EIS Fitting in the Supporting Information, in particular the equivalent circuit in Figure SI.8 and the calculated values in Table SI.1. The CPE is necessary to take into account the depression of the semicircles; in particular, the HF process is markedly depressed. Both of the processes are observed to be influenced by pH_2_, so they are related with the anodic compartment. The behavior of the HF process is not completely linear. With increasing temperature, it shifts toward lower frequencies and is more and more depressed in a tendency to disappear in the adjacent peak. However, at 750 °C, its peak phase increases again, with the relaxation frequency continuing the trend toward lower frequencies. With increases in pH_2_, the frequency still decreases. The prominence of the peak decreases too, apart from 20% H_2_, which is higher than 10% H_2_.

**Figure 5:**
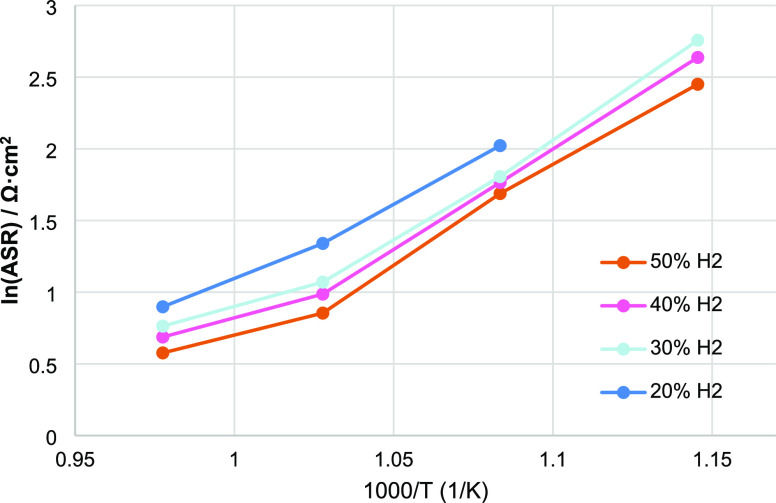
Arrhenius plot of ASR variation depending on temperature and concentration of hydrogen.

The capacitance of this process is around 10^–6^–10^–7^ F/cm^2^, it is thermally activated, and its apex frequency is between 10^4^ and 10^5^ Hz; all of these results match with a charge transfer process at the anode electrolyte interface.^60^ The dependence on pH_2_ is not always observed for this process, and when it is, it is in general associated to chemical modifications on the interface induced by pH_2_ and local formation of new phases.^61^ The LF process is thermally activated, favored by a higher pH_2_, and its relaxation frequency increases with temperature and pH_2_. All of these features match for the dissociative adsorption of hydrogen,^62^ which is favored by both the temperature and high pressure of hydrogen. ASR values obtained by fitting are shown in Figure 5 as logarithms. The plots are linear up to 700 °C, but for all the concentrations of hydrogen, at 750 °C, the pendency changes. This indicates a noticeable variation in the activation energy toward lower values, from 117 kJ/mol below 750 °C to 46 kJ/mol above that temperature. Such changes are often correlated with chemical modification of the catalyst, for example, phase transitions^63^ or radical changes in limiting steps of the process, and it could be interesting to find if it is possible to extend the low activation energy regime also to lower temperatures.

#### MnO_x_/LSGF|LSGM|LSGF/FeO_x_ Full Cell

Hereby, complete cells were tested using alternative anode material, which we have already studied in the first part of this work.^26^ The good performance of the cathode FeO_x_/LSGF was attributed to the formation of a thin Sr/Fe-rich foil on the surface of the electrode during SOFC thermal treatment; this very thin foil significantly improves the behavior of the electrode (as observed by comparison with LSGF). The electrochemical results are encouraging for future applications in SOFCs because the nanocomposite has an ASR of 2.1 Ωcm^2^ at 620 °C, and only one-third of that of the LSGFs under the same conditions. All the following results were collected using the full cells: MnO_x_/LSGF|LSGM|LSGF/FeO_x_. Because of the well-known activity of Mn-based materials toward carbon-containing fuels, the MnO_x_/LSGF anode was evaluated using propane beyond the most common hydrogen.

**Figure 6:**
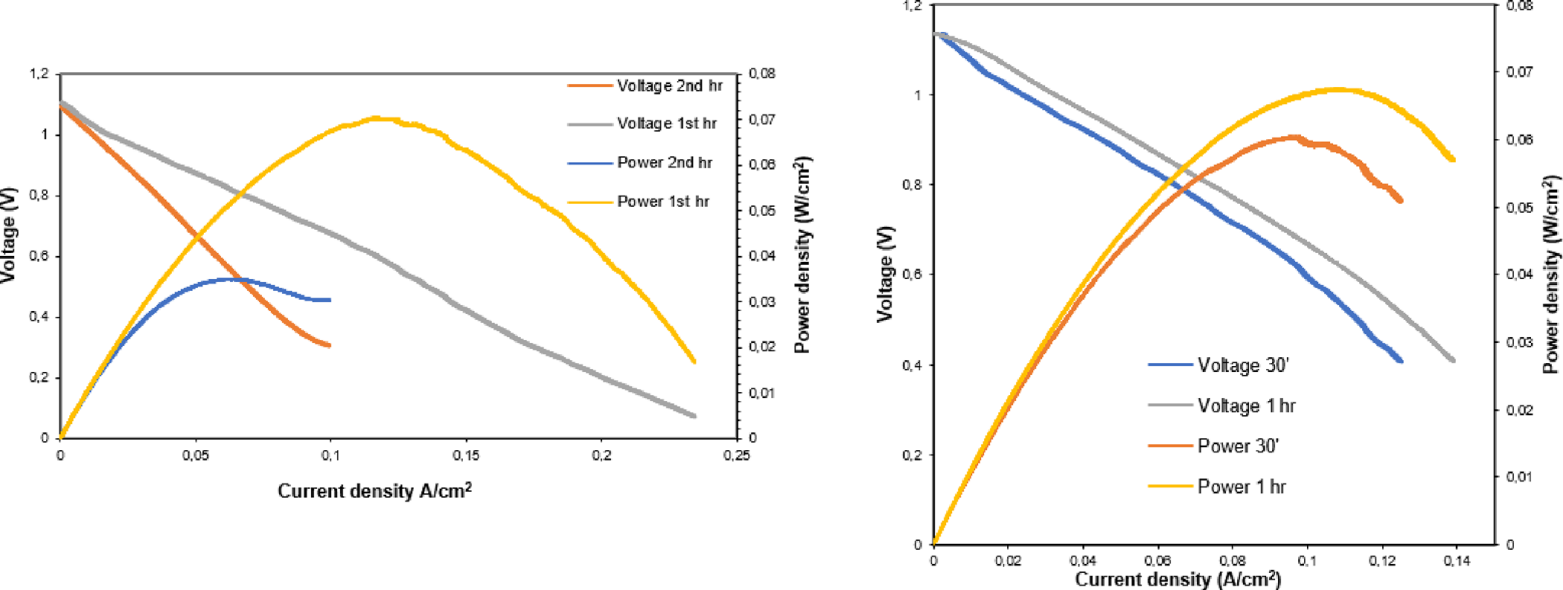
Left: cell polarization curves after switch-on (first hour) and after 1 h of operation (second hour) at 744 °C. The anode is fed with pure H_2_, 53 sccm. Right: polarization curves and power of the cell fed with propane at the anode. Results after 30 min and 1 h of operation are reported. The temperature is 744 °C, and the gas flow at the anode is 50 sccm. In both cells, the cathode was fed by air.

First of all, the complete cell was tested in hydrogen and the performances are shown in Figure 6, left. After 1 h of operation, the maximum power decreases from 70 to 35 mW/cm^2^. The OCV was excellent, around 1.10 V, and it appears not to be affected by the decrease in performances from the first to the second hour. After the testing of several cells based on LSGF, it can be easily noticed that optimal OCVs have always been recorded. The minimal voltage losses must be attributed to the LSGM electrolyte because of its pure O^2–^ ion conductivity, but the electrode material ability to carry on this reaction with only minimal voltage losses should not be ignored. This is promising as cells with high OCVs tend to be more efficient.

Feeding of the cell with propane gave remarkable results. As shown in Figure 6, right, the current and power increase, compared to hydrogen feeding, and an output of 67 mW/cm^2^ was reached again. The OCV had even increased from hydrogen feeding and had reached 1.13 V. No short-term performance depletion was observed when propane was used as a fuel. Impedance spectra (Supporting Information, Figure SI.7) were composed of the same arcs of hydrogen feeding, plus a large signal at very low frequencies, which stands for a high gas-diffusion resistance.^64^ Its magnitude suggests that a better optimization of porosity of the electrode could greatly improve the performances with this fuel. Performances under propane are interesting because the difficulty to use hydrocarbon-based fuel with common nickel-based anodes is one of the biggest flaws of current SOFCs.^65^ The finding of an efficient anode material, able to work smoothly also with carbon-containing fuel, would open considerable opportunities for the SOFC market. This result suggests that LSGF + MnOx could be an interesting candidate for this task; however, some improvements in the material can be studied. For example, to increase the electronic conductivity of the supported LSGF powder to vary the stoichiometry of species in the B-site may be a solution. In LSGF, the electronic conductivity is driven by redox couples Fe^2+^/Fe^3+^ and Fe^3+^/Fe^4+^ and then increasing their content would enhance the electronic conductivity and in general the electrochemical performances of the cell. To solve this task is possible to increase the content of iron replacing gallium or varying the ratio between La and Sr in the A-site. Another solution could be to reduce the thickness of the LSGM electrolyte (in this work, 1.2 mm), decreasing in this way the ohmic losses.

**Figure 7:**
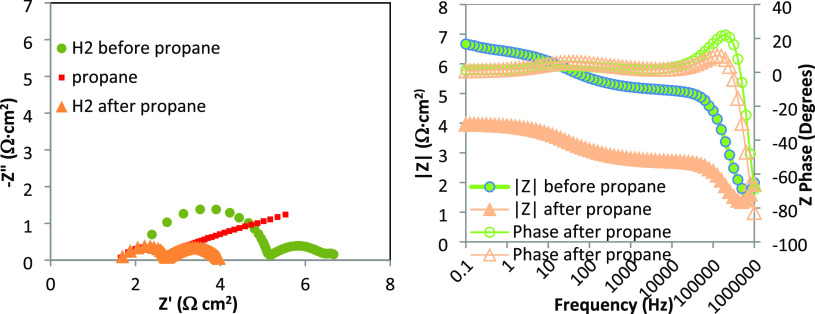
Comparison between impedance spectra before, during, and after propane feeding. Left: Nyquist plots. Right: Bode plots.

Propane does not only induce good performances but also proves to be beneficial for the electrode. Comparing between impedance spectra before and after propane (Figure 7), it is evident that the change is mainly due to the high frequency component, which was related to electrode/electrolyte interfacial charge transfer, while the low frequency arc is unaffected. Changes in interfacial charge transfer depending on the atmosphere are possible; they often have been related to the local formation at the interface of specific phases, able to impede, in case they are insulators, the passage of O^2–^ ions from the anode to the electrolyte. It is possible that something similar has happened also in this case, but specific analysis on the interface should be carried out to assess it. The cause of the improved connection between the anode and electrolyte is reversible (and this is coherent with the formation of new phases and interface) as this effect is partially lost after some hours of operation.

## Conclusions

A new anode material, MnO_x_ on LSGF, has been synthesized by wet impregnation on supported perovskite, and it has been largely characterized and tested for fuel cell operation. LSGF is able to absorb manganese during heating until 1000 °C but releases it at 1100 °C and during cooling. It has been demonstrated that manganese oxide and consequent temperature treatment affects the cation segregation of the supported LSGF perovskite. EIS measurements were analyzed by Nyquist and Bode curves and two processes have been identified. The high frequency process has been attributed to charge transfer at the electrode/electrolyte interface and that at low frequency derives from the dissociative adsorption of hydrogen. The Arrhenius plot of ASR suggests two different trends, and the activation energy decreases from 117 kJ/mol at 750 °C to 46 kJ/mol above that temperature. This change can be often explained by chemical modification of the catalyst or variation in different processes involve. A cell entirely based on La, Sr, and Ga perovskites, designed to attain a great chemical stability, has been tested in hydrogen and propane, reaching 70 and 67 mW/cm^2^, respectively. Different strategies to improve performances have been outlined. In general, the cell showed optimal OCVs and very promising performances under propane. This makes the anode MnO_x_/LSGF a suitable candidate for operation with carbon-containing fuels.

## Supporting Information Available

XRD vs temperature patterns of LSGF + 10% mol MnO_x_ including formation of all phases during thermal heating; XRD measurements after H_2_-TPR analysis are also provided; SEM images before and after deposition of 10% MnO_x_ on LSGF are provided using different magnification in order to point out the effects of the deposition on the morphology; N_2_ isotherm of LSGF and LSGF + MnO_x_ including the explanation of the graphs; examples of the fitting of the XPS spectra of O 1s and Mn 2p_x_ + LSGF|LSGM|LSCF as a function of both the temperature and hydrogen content; and Nyquist plots of MnO_x_ + LSGF|LSGM|LSGF + FeO_x_ collected at 744 °C under a flow of pure hydrogen and pure propane; deeper investigation of EIS spectra and fitting results were provided. (PDF)
