# Supplementary material for: Rational Development of IT-SOFC Electrodes Based on the Nanofunctionalization of La0.6Sr0.4Ga0.3Fe0.7O3 with Oxides. Part 2: Anodes by Means of Manganite Oxide
Source: ACS Appl Energy Mater. 2022 Dec 28;6(1):141–50. doi: 10.1021/acsaem.2c02592 (PMC9832435; doi:10.1021/acsaem.2c02592)
Supplement: Supplementary file 1 — ae2c02592_si_001.pdf [file ae2c02592_si_001.pdf]

Supporting Information

Rational Development of IT-SOFC Electrodes Based on the Nanofunctionalization of $La_{0.6}Sr_{0.4}Ga_{0.3}Fe_{0.7}O_3$ with Oxides. Part 2: Anodes by Means of Manganite Oxide

Jonathan Cavazzani,*,† Andrea Bedon,† Giovanni Carollo,† Mathilde Rieu,‡ Jean-Paul Viricelle,‡ Antonella Glisenti †, §

†*Department of Chemical, Sciences, University of Padova, Via F. Marzolo 1, 35131 Padova, Italy*

‡*Mines Saint-Etienne, Univ. Lyon, CNRS, UMR 5307 LGF, Centre SPIN, F – 42023 Saint-Etienne, France*

§*ICMATE - Department of Chemical Sciences, University of Padova, Via F. Marzolo 1, 35131 Padova, Italy*

Corresponding author: Jonathan Cavazzani
e-mail: jonathan.cavazzani@phd.unipd.it
Tel: +39 (0)049 827-5858

XRD.

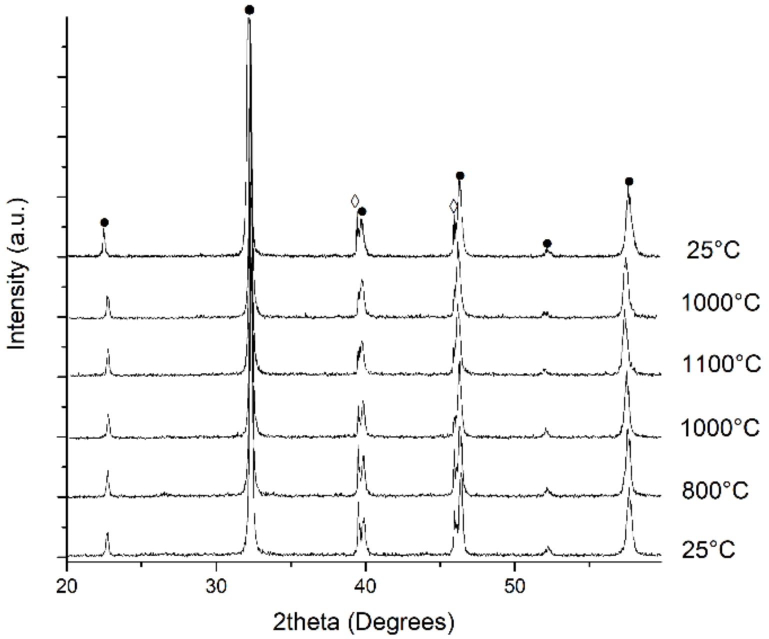

Figure SI.1. *XRD patterns of LSGF + 10% MnO_x. Composite, treatment from 25°C (lowest pattern) to 1100°C and then back to 25°C (highest pattern). solid circles: LSGF, rhombohedral, JPCDS 04-016-7460; open diamonds: Pt (substrate), cubic, JPCDS 00-004-0802.*

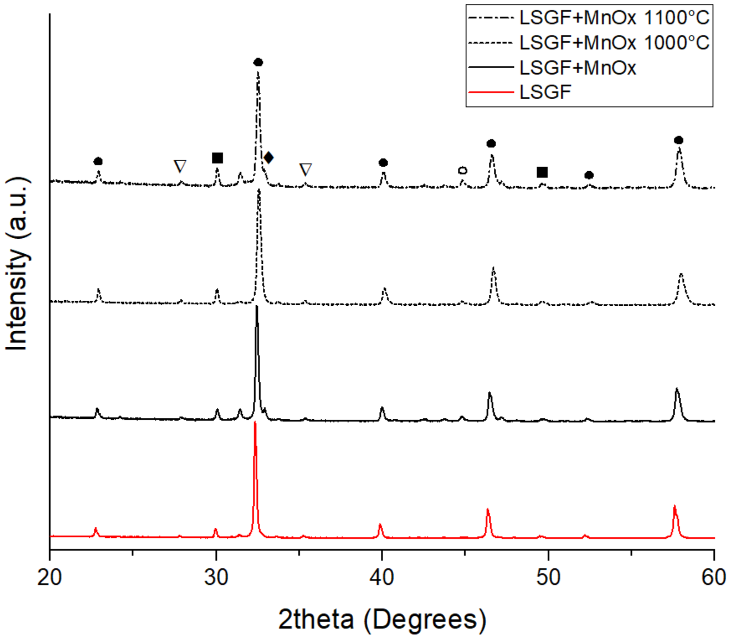

Figure SI.2. *XRD patterns of LSGF and LSGF + 10% MnO_x post TPR analysis. solid circles: LSGF, rhombohedral, JPCDS 04-016-7460; open inverted triangles: Mn_3O_4, hausmannite, tetragonal, JCPDS 04-007-1841; solid diamonds: Mn_2O_3, bixbyte, cubic, JPCDS 00-041-1442; open diamonds: Pt (substrate), cubic, JPCDS 00-004-0802; open circles: Fe, 96-500-0218; ▪: La_2O_3, 96-101-0279.*

SEM on powders.

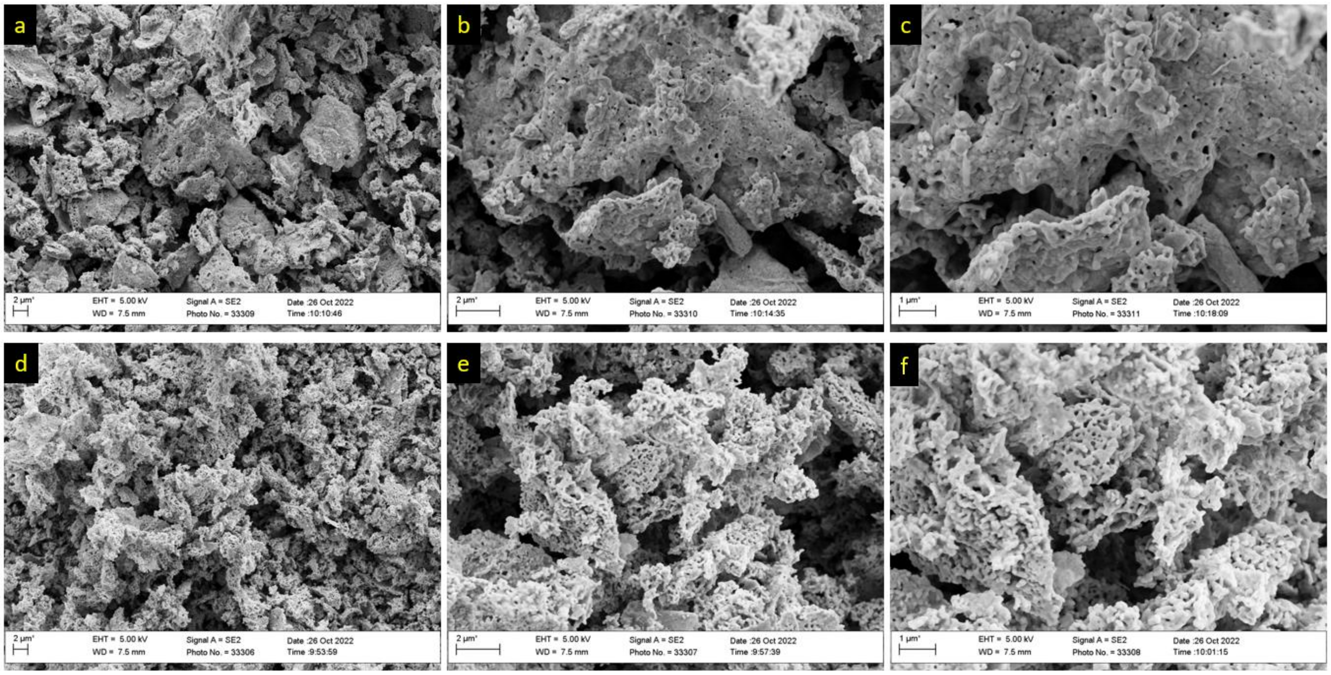

Figure SI.3. *SEM images before and after deposition of manganese oxide; a, b and c: SEM images collected at different scale of LSGF powder; d, e, f: SEM images collected at different scale of LSGF + 10% MnO_x treated at 550°C.*

SEM images obtained before and after the deposition of the Mn oxides exhibit different morphology, Figure SI.3. In particular, in Figure SI.3 (a, b, c), SEM images of the LSGF powder show the presence of relatively large aggregates of LSGF perovskite. After the MnO_x deposition, there are no changes on the supported powder (LSGF) morphology, however brighter clusters of MnO_x of smaller size were observed, Figure SI.3 (d, e, f). There are well-dispersed on the surface, indeed they are not detected by XRD measurements using a deposition of 10% of MnO_x (Figure SI.1). In addition, images suggest the porosity as well as Specific Surface Area (SSA) increase. Indeed, BET measurements (see N_2 isotherms, Figure SI.4) results in an increment of SSA from pure LSGF (4.4 m^2/g) to MnO_x/LSGF (15.9 m^2/g).

N_2 isotherms.

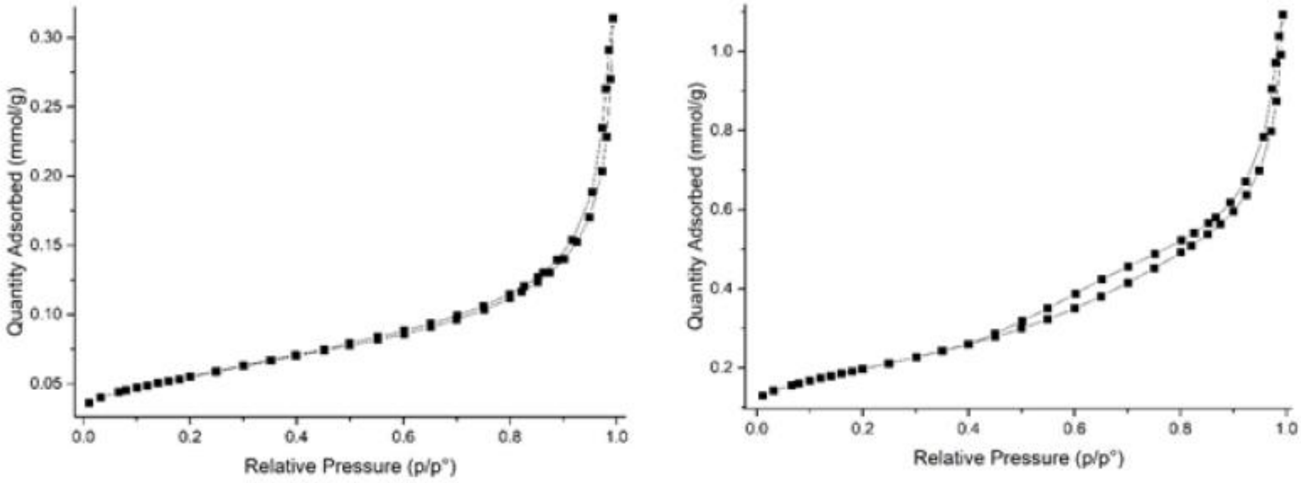

Figure SI.4. *Left: N_2 isotherm of LSGF. Right: N2 isotherm of LSGF + MnOx.*

The comparison of the N_2 isotherms of LSGF and of the MnOx/LSGF (Figure SI.2) indicates a strong modification of the powders after the impregnation process, as expected in case of a good deposition of the oxides on the perovskitic substrate. Superficial area increases, from 4.4 m^2/g measured for the pure LSGF perovskite, to 15.9 m^2/g for the MnOx/LSGF. The shape of the isotherms differs suggesting a different morphology. The LSGF perovskite shows a type II isotherm, along with a very low superficial area. The deposition of the oxide causes, beyond the increase in the superficial area, a transition of the isotherm towards type IV, that can be related to the formation of mesoporosities in which adsorbed N_2 gas can condensate leading to the typical hysteresis [1]. In MnOx/LSGF the curve still resembles type II with a principle of hysteresis. The hysteresis can be classified as type H3, which is associated to the presence of slit-shaped pores [2].

XPS.

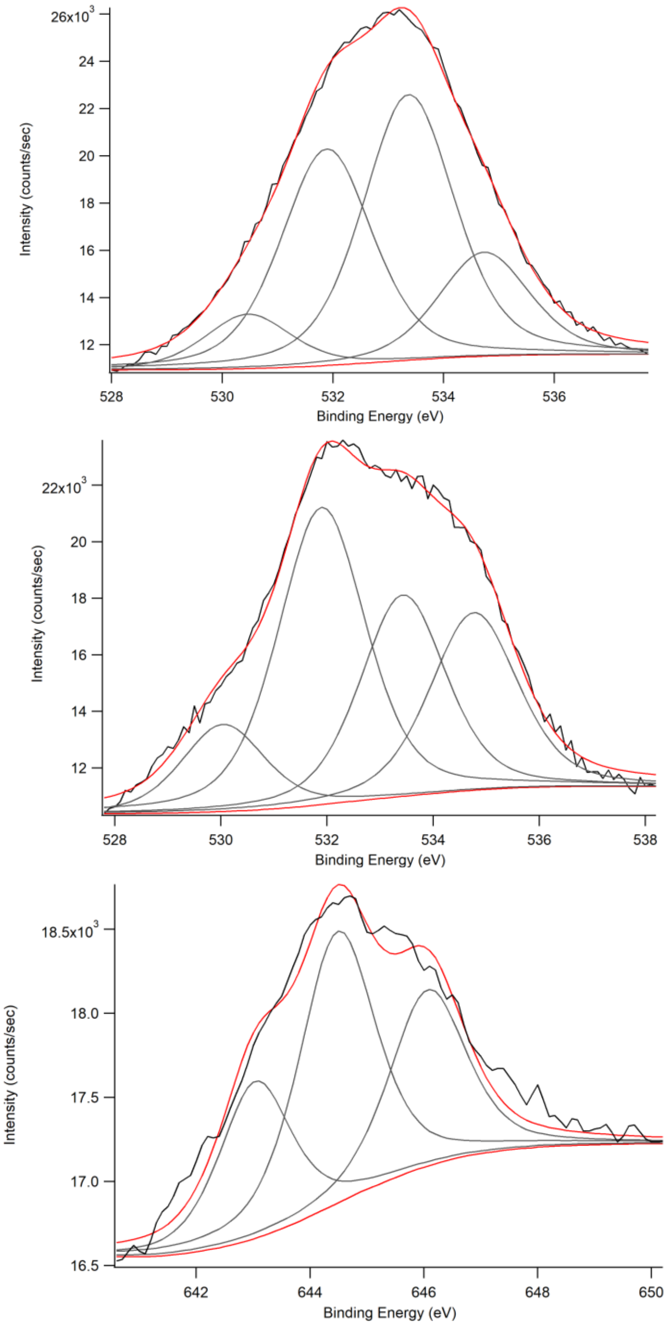

Figure SI.5. *Examples of the fitting of the XPS spectra of O 1s (upper LSGF+MnOx, middle LSGF+MnOx treated at 1100°C), and (lower) Mn 2p3/2 peak of LSGF + MnOx.*

Cell Measurements. *MnOx/LSGF|LSGM|LSCF full cell.*

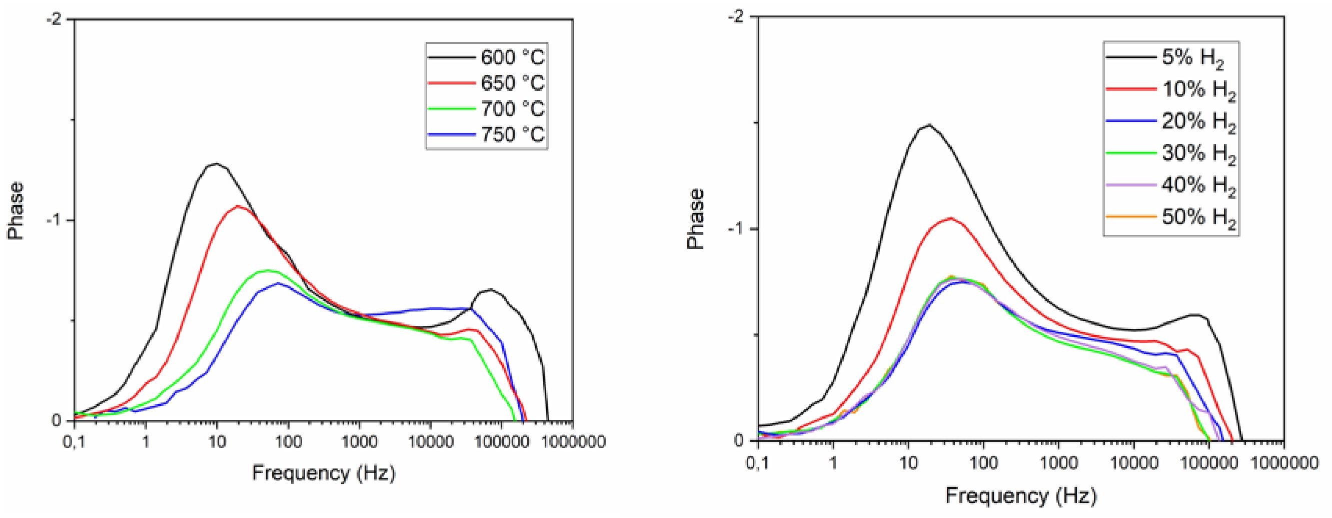

Figure SI.6. *Bode plots of the full cell MnOx/LSGF|LSGM|LSCF are reported as function of temperature using 30% of hydrogen (left) and as function of hydrogen content at 700°C (right).*

Cell Measurements. *MnOx/LSGF|LSGM|FeOx/LSGF full cell.*

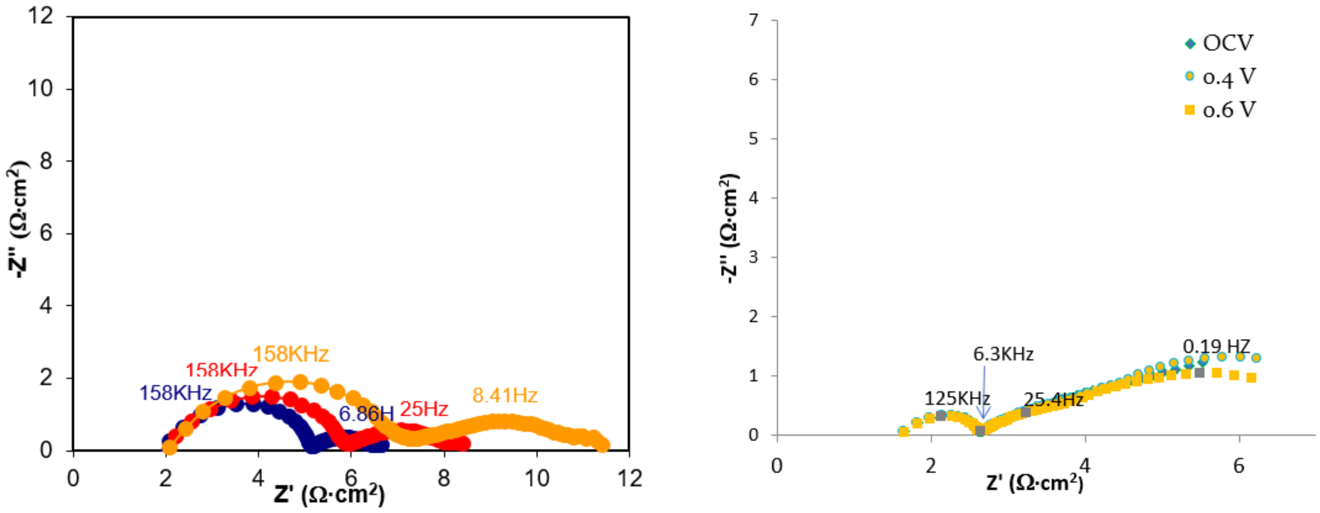

Figure SI.7. *Left: Nyquist plots of MnOx/LSGF|LSGM|FeOx/LSGF collected at 744°C, anode fed with pure hydrogen, flow 53 sccm. Blue = OCV, Red = 0.6 V, Yellow = 0 V. Right: Impedance spectra of cell fed with propane at different voltages, after 1 hour of operation. Temperature is 744°C, propane flow is 50 sccm.*

EIS Fitting

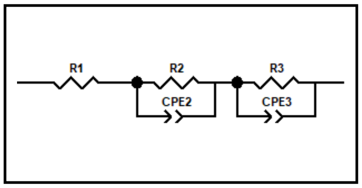

Figure SI.8. *Equivalent circuit used to describe the EIS spectra.*

All EIS spectra in Figure 4 were fitted by the same equivalent circuit, already studied in literature to describe the LSGF -based materials, reported in Figure SI.8 [3]. It is made up by a resistor R1 used to take into account the resistance of the electrolyte and two R_x-CPE_x. The process at higher frequency can be assigned to a charge transfer process, while the process at lower frequency can be attributed to a dissociative adsorption of hydrogen. Others details are discussed in the manuscript.

In Table SI.1 all values obtained by EIS fitting are reported in Table SI.1. They were calculated by ZView software. The capacitance for Constant Phase Element (CPE) were calculated as described by the literature [4].

Table SI. 1. EIS fitting results and parameters [a]

**Temperature (°C)**	**ASR_2 (Ω cm^2)**	**C_2 (µF)**	**ASR_3 (Ω cm2)**	**C_3 (µF)**
600	156.9	2.4	143.1	0.5
650	63.9	2.9	60.0	0.5
700	31.4	2.6	31.9	1.7
750	27.1	2.0	19.2	2.1
**H_2 Content (%)**	**ASR_2 (Ω cm^2)**	**C_2 (µF)**	**ASR_3 (Ω cm2)**	**C_3 (µF)**
5	48.3	0.2	142.7	1.3
10	35.5	0.1	70.8	2.0
20	22.9	4.0	59.1	1.4
30	18.1	7.8	46.9	1.3
40	13.1	0.2	42.8	2.6
50	10.4	2.6	36.0	1.3

[a] *Data were calculated using the equivalent circuit reported in Figure SI.8 by the software ZView.*

References

(1) Gregg, S. J.; Sing, K. S. W.; Salzberg, H. W. Adsorption Surface Area and Porosity. *J. Electrochem. Soc.* **1967**, *114* (11), 279C. 10.1149/1.2426447.

(2) ALOthman, Z. A. A Review: Fundamental Aspects of Silicate Mesoporous Materials. *Materials (Basel).* **2012**, *5* (12), 2874–2902. 10.3390/ma5122874.

(3) Ayachi, S.; Moualhi, Y.; Rahmouni, H.; Gassoumi, M.; & Khirouni, K. Chromium concentration effects on transport and dielectric behaviour of lanthanum-gallium ferrite. *Physica B: Condensed Matter*, **2020**, *591*, 412244. 10.1016/j.physb.2020.412244

(4) Irvine, J. T.; Sinclair, D. C.; West, A. R. Electroceramics: characterization by impedance spectroscopy. *Advanced materials*, **1990** *2*(3), 132-138. 10.1002/adma.19900020304
